# Combating Sale of Counterfeit and Falsified Medicines Online: A Losing Battle

**DOI:** 10.3389/fphar.2017.00268

**Published:** 2017-05-16

**Authors:** Kah Seng Lee, Siew Mei Yee, Syed Tabish Razi Zaidi, Rahul P. Patel, Quan Yang, Yaser Mohammed Al-Worafi, Long Chiau Ming

**Affiliations:** ^1^Unit for Medication Outcomes Research and Education, Pharmacy, School of Medicine, University of TasmaniaHobart, TAS, Australia; ^2^Faculty of Pharmacy, SEGi UniversitySelangor, Malaysia; ^3^Department of Pharmacy and Pharmacology, University of BathBath, UK; ^4^Clinical Pharmacy Department, College of Pharmacy and Health Sciences, Ajman UniversitySharjah, UAE

**Keywords:** fake medicines, falsified medicines, substandard drug, illicit drug, medicines trafficking, prescription medicines

## The lucrative and popularity of selling medicine online

The rapid growth of technology has transformed many brick-and-mortar businesses into online businesses, and medicines are now being sold over the internet. Influenced by the notions that online purchases are economical and do not require a prescription, the general public are keen to purchase medicine online through websites, social media and mobile apps. Online medicine purchase is presumed to be convenient and confidential, free from embarrassment of sharing personal and sensitive health information to a healthcare professional. Public in United States, Europe, Australia is generally aware that internet sales form part of the official medicines distribution channels, often a valid prescription is required for controlled medicine. However, unlicensed, substandard and falsified medicines with various dubious medical claims are advertised and sold illegally in many rogue online pharmacies (Jack, 2016). These include medications for weight loss, hair growth, and treatment of erectile dysfunction. Such medicines are termed as substandard, spurious, falsely labeled, falsified and counterfeit medical products by the World Health Organisation (WHO). Similarly, the European Commission defines such products as falsified medicines or fake medicines that pass themselves off as real, authorized medicines (European Commission, 2016). These medicines may contain substandard active ingredients, which are low quality and/or an incorrect amount, either too high or too low, and have not been properly evaluated by authorities in terms of quality, safety, and efficacy. It must be noted that falsified medicines are often confused with counterfeit medicines. According to European Commission, counterfeit medicines refers to medicines that do not comply with European Union law on intellectual and industrial property rights, for example, unregistered medicines sourced from parallel import (European Medicines Agency^1^). In this article, the illegal sales of both counterfeit and falsified medicines (CFMs) are discussed.

In 2012, the WHO estimated the CFMs industry to be worth USD 431 billion a year, but further estimates has not been reported in the recent years due to the fast growing, widespread practice of this industry, making it impractical to estimate on a global scale (Garrett, 2012). Authorities are finding it difficult to curb CFMs due to the lack of governance over the internet. Furthermore, fragmented cybercrime legislation leads to large substantive and procedural lacunae in law, rendering law enforcement efforts useless.

## Plaguing the lacunae: case studies of India, China and African Countries

The crackdown on CFMs sale by authorities can only be effective with the present of relevant legislation to empower the judiciary to impose substantial sentences. Countries such as China, India and African countries previously had either no specific or weak law governing the control of CFMs. To address this problem, new legislations have been drafted and combined with the existing legislation. Strengthening of drug legislation is vital as CFMs are found alongside genuine medicines in legitimate distribution channels, making it impossible for consumers to determine the authenticity of the medicines (Office of the United States Trade Representative, 2016). In 2013, the China Food and Drug Administration led an inter-agency operation named “Two Strikes, Two Setups” targeting illegal manufacture and sale of medicines, which successfully closed down 194 Chinese websites and reported 609 overseas websites to their relevant countries for further enforcement actions (Alliance for Safe Online Pharmacies, 2015). China, believed to be the biggest producers of CFMs, is showing encouraging signs in this issue with the amendment of Article 141 of the Criminal Law in 2011. The amended penalties for manufacturing and selling CFMs are punishable to a minimum imprisonment of 10 years, in addition to a fine or forfeiture of property (Congressional-Executive Commission on China, 2011). For India, harsh sentences imposed in 2008 through the Drug and Cosmetic Act 1940 amendments, result in a minimum 10 years and/or a minimum fine of Indian Rupees (INR) 1 million (USD 15,000) or three times the value of the medicines confiscated. However, the anti-counterfeiting and anti-falsifying provision does not extend to the regulating of online pharmacies, due to the lack of clarity in the Drugs and Cosmetics Act 1940 and the Drugs and Cosmetics Rules 1945 (Nagaraj et al., 2014). In December 2015, the office of the Drugs Controller General (India) issued a directive banning the sale of medicines over the internet and taking action against online pharmacies such as Zigy.com, Netmeds.com and mCHEMIST.com (Reddy, 2015). However, in the absence of clear legislations and policies on online pharmacies, the directive is under scrutiny due to the differences in the interpretation of law, impeding the authorities' efforts to curb CFMs sold in illegal online pharmacies.

In Africa, the East African countries such as Uganda and Kenya are strengthening and enforcing laws against CFMs. Uganda's new Anti-Counterfeiting Goods Bill, passed in October 2015 aims to introduce punitive, deterrent and effective measures for combating the production or marketing of counterfeit goods. Prior to 2015, there were no law that prohibits or controls the manufacturing and marketing of counterfeit and falsified goods. Although the National Drug Policy and Authority Act 1993 provides some guidance on the sale and supply of substandard medicine, the non-specificity of this act on CFMs is seen as the failure of Uganda war against the offense. In addition, a minimum jail term (7 years) was introduced in the new bill, a penalty absents from the National Drug Policy and Authority Act (Parliament Watch, 2015). In Kenya, efforts against CFMs have been strengthened significantly over the past few years, with the establishment of the Anti-Counterfeit Act in 2008 and the passing of several amendments. The latest amendment in 2014 paved the way for the establishment of an Intellectual Property Enforcement and Co-ordination Advisory Committee and the power to compound offenses, reducing the hassle and resources of prosecution and conviction through the judiciary.

Nevertheless, the efforts of these countries in strengthening pertinent legislation are inadequate with the growing widespread availability of CFMs over the internet. This global public health threat extends beyond national borders and many countries currently do not have specific laws that deal with the selling of CFMs over the internet (Govtrack.us, 2008).

## Weaning impact of international collaborative operations

Because of extensive intermediaries' and suppliers' networks across the globe, the sale of CFMs through illegal online pharmacies is difficult for law-enforcement agencies to control. Such organized crimes mostly operate using rogue domain name registrars, electronic payment systems and international and local delivery services. Standalone enforcement efforts by individual countries have failed to cripple the networks, only temporarily ceasing operations in these countries before they became fully re-operational after a few days. Seeing the need for an integrated enforcement operation, INTERPOL along with other agencies launched Operation Pangea, a global cooperative operation targeting the online sale of CFMs. During the operations, the makers and distributors of CFMs are identified and these medicines are removed from the supply chain (INTERPOL, 2016).

In 2016, Operation Pangea IX targets 3 main aspects of illicit medicine trafficking through the Internet Service Providers (ISPs), payment systems and electronic delivery services. The operation also targets some main aspects that are exploited by organized crime in trafficking medicines online: fraudulent domain name registrars, electronic payment systems and medicine delivery. The success in these operations (Table 1) is also because of the involvement of private internet-related agencies and agencies that handle online payments. These agencies such as MasterCard, PayPal and VISA are crucial to curtailing financial support (INTERPOL, 2016). However, Operation Pangea is slowly showing signs of over-inflated impact as it is an intrinsic limitation to check enormous quality of shipments from all custom checkpoints regularly and effectively.

**Table 1:** **Results of operation pangea I till IX (2008 till 2016) (INTERPOL, 2016)**.

**OP/Year**	**Countries participated**	**Agencies involved**	**Websites suspended**	**Suspects arrested/ under investigation**	**Number of pills seized (millions)**	**Value of seized product (USD millions)**	**Postal packages seized**
OP IX/2016	103	193	4,932	393	12.2	53	170,340
OP VIII/2015	115	236	2,410	156	20.7	81	NA
OP VII/2014	113	198	11,800	434	9.6	32	NA
OP VI/2013	99	NA	13,700	213	10.1	36	41,000
OP V/2012	100	NA	18,000	80	3.75	10.5	6,700
OP IV/2011	81	NA	13,500	55	2.4	6.3	8,000
OP III/2010	44	NA	297	87	2	6.77	NA
OP II/2009	25	NA	153	59	NA	NA	NA
OP I/2008	10	NA	NA	NA	NA	NA	NA
Mean	77	209	8,099	185	9	32	56,510
Median	99	198	8,366	122	10	32	24,500

*OP, Operation Pangea; NA, Not available*.

## Way forward: raising international collaboration and consumer awareness

Due to the sheer size of organized crime networks, stand-alone operations in individual countries might not be adequate to stop the overall trend. Concerted inter-country and inter-regional operations may well be the answer to this conundrum. The Alliance for Safe Online Pharmacies EU (ASOP EU), formed in 2011, is a coalition dedicated to protecting patient safety online. Currently ASOP EU has 31 members including Google, Pfizer and EAASM, as well as 26 observers including eBay, PayPal, Visa and Microsoft (Alliance for Safe Online Pharmacies EU, 2016). Regional partnerships such as ASOP EU and EAASM are important to exert influence on the political and legislative bodies to ensure new regulation and regular enforcement of online surveillance. Similarly, these alliances could strengthen collaboration among law-enforcement agencies on cybercrime involving pharmaceuticals and strengthen the human resource and capacity to tackle cybercrime.

As a follow-up to the successful piece of research “The Counterfeiting Superhighway,” EAASM launched an innovative “Counterfeiting the Counterfeiter” campaign in Germany in 2011 to increase public awareness about CFMs sold online and direct patients to safe and legitimate sources of medicines at the same time. In this project, several websites were designed to attract unsuspecting regular users to login to order their medications without having a prescription. Immediately, when the users clicked the purchase button, they were directed to an authorized online pharmacy website which gave an educational message about the risk of illegal websites. An average of 2,800 people browsed the disguised websites each day and the projected sales volume (based on items intended for purchase) was USD 13.2–38.6 million per year. This study confirmed the hypothesis of the EAASM that illegal online pharmacies have low start-up costs and exorbitant returns (European Alliance for Access to Safe Medicines, 2012).

The Falsified Medicines Directive was introduced by the European Commission in 2013. It entails a certification mark or common logo that is compulsory for websites selling medicinal products to display. The certification mark will enable patients to differentiate authorized online medicine retailers. These authorized online retailers must provide a link on their webpage to the national pharmaceutical regulator's website to ensure potential customers can double-check the authenticity of the legal status of the operator.

Emphasis must be given to raise consumer awareness about the risks and dangers of buying medicines online. The Medicines and Healthcare products Regulatory Agency (MHRA) confirmed that 79% of the UK public who took part in a survey on online medicine purchases were unaware of CFMs (Medicines Healthcare Products Regulatory Agency, 2016). As a result, the MHRA is creating public awareness about CFMs sold online via a campaign webpage with practical tips on recognizing legitimate online retailers of medicines and medical devices (Medicines Healthcare Products Regulatory Agency, 2016). The Food and Drug Administration (FDA) in the US has embarked on a similar campaign “BeSafeRx: Know Your Online Pharmacy” to disseminate information about how to identify an illegal pharmacy website and cues to detect illegal websites (U.S. Food Drug Administration, 2016). It must be noted that the public is advised to log on to the regulatory authorities' portals to check for the details of the medicine approvals as well as licensing. A country-by-country list of the websites to check the medicine registration information is available at http://crash2.lshtm.ac.uk/Regulatory.htm. Online medicine retailers should always adhere to the provisions of existing laws because selling medicines in cyberspace does not mean they are outside of the law. The public is also advised to be vigilant when buying medicines online and should always make checks before clicking to purchase.

## Conclusion

To date, health authorities' efforts in combating CFMs by strengthening the anti-counterfeit and anti-falsifying legislations should be lauded. However, immediate action needs to be taken to fill in the legislative lacunae of the online sale of CFMs. In addition, the international collaboration against the online sale of CFMs should be continued by encouraging more countries to participate. Nevertheless, the fight against CFMs should not be limited to legislation, enforcement and global collaboration of government authorities *per se*. Instead, multipronged strategies, including adoption of anti-falsifiying technology and raising the awareness among all stakeholders, principally the general public may be the turning point in winning the battle against this unscrupulous business.

## Author contributions

Conceived and designed the experiments: RP, SZ, LM. Wrote the paper: KL, SY, RP, SZ, LM. Designed search strategies: KL, SY, RP, SZ, QY, YA, LM. Critically reviewed the manuscript for important intellectual content: KL, SY, RP, SZ, LM. All authors read and approved the final version.

### Conflict of interest statement

The authors declare that the research was conducted in the absence of any commercial or financial relationships that could be construed as a potential conflict of interest.

## Footnotes

^1^European Medicines Agency Falsified medicines. Available from: http://www.ema.europa.eu/ema/index.jsp?curl=pages/special_topics/general/general_content_000186.jsp. Accessed 21 March 2017.
